# Detection of human cytomegalovirus in motile spermatozoa and spermatogenic cells in testis organotypic culture

**DOI:** 10.1186/2042-4280-2-7

**Published:** 2011-06-28

**Authors:** Victor A Naumenko, Yurii A Tyulenev, Sergei A Yakovenko, Lubov' F Kurilo, Ludmila V Shileyko, Aleksander S Segal, Larisa E Zavalishina, Regina R Klimova, Anton S Tsibizov, Sergei V Alkhovskii, Alla A Kushch

**Affiliations:** 1 The D. I. Ivanovsky Institute of Virology, Ministry of Health and Social Development of the Russian Federation, 123098 Gamaleya str. 16, Moscow, Russia; 2 Altravita IVF Clinic, 117186 Nagornaya str. 4a, Moscow, Russia; 3 Medical & Genetic Research Center of Russian Academy of Medical Sciences, 115478 Moscworechye str. 1, Moscow, Russia; 4 Department of Urology, Moscow State Medical Dental University, 127473 Delegatskaya str. 20/1, Moscow, Russia; 5 P.A. Herzen Research Oncological Institute, 125284 2nd Botkinsky pr. 3, Moscow, Russia

**Keywords:** human cytomegalovirus, infertility, spermatogenesis, testis organotypic culture

## Abstract

**Background:**

The presence of human cytomegalovirus (HCMV) in male genital tract suggests its vertical transmission with spermatozoa and the development of a potentially dangerous fetal infection. The objective of the present study was to evaluate the possibility of intracellular HCMV localization in male germ cells and to examine the effect of the virus on human spermatogenesis.

**Methods:**

Semen samples from 91 infertile and 47 fertile men were analyzed. HCMV was detected by real time PCR, rapid culture method and PCR in situ. Human testis organotypic culture and quantitative karyological analysis were used to investigate viral effects on spermatogenesis. Localization of HCMV in immature germ cells and spermatozoa was studied by immunostaining with monoclonal antibodies and ultrastructural analysis of infected organotypic culture.

**Results:**

Viral DNA was detected in 12.3% samples of motile spermatozoa, while infectious activity only in 2.9% infertile and fertile men without statistically significant intergroup difference. According to PCR in situ, the mean percentage of infected cell in both groups was 1.5% (0.25%-15%), which can serve as a criterion for evaluating the risk of HCMV transmission. In HCMV-infected organotypic culture viral antigens were identified in spermatides on day 4, in spermatogonia and spermatocytes on day 8, and in spermatozoa on day 14. Empty and full capsides and virions were visualized in germ cells by electron microscopy. The number of cells before introduction in culture was taken for 100%. On day 14 infected culture contained 36.8% spermatogonia, 18.7% spermatocytes, 27.6% round spermatides and 42.5% elongated spermatides; in comparison with 82.2%, 51.5%, 70.4% and 65.7% in uninfected culture, respectively (all p < 0.05). There were no changes in the number and viability of spermatozoa.

**Conclusions:**

HCMV was detected in male germ cells, both in sperm samples and in testis organotypic culture. The virus may infect immature germ cells which develop to mature HCMV-carrying spermatozoa. A considerable decrease in the number of immature germ cells indicates that HCMV produces a direct gametotoxic effect and can contribute to male infertility.

## Background

Male infertility accounts for 20-50% infertile couples and is often associated with genital infections [1]. Negative effects on reproductive function have been proposed for such viruses as the human immunodeficiency virus, human papillomavirus, herpes simplex virus, Epstein-Barr virus [1-4]. Human cytomegalovirus (HCMV) is widespread in human population and can be transmitted sexually. The effects of HCMV on spermatogenesis and its vertical transmission with sperm cells have not been investigated in sufficient detail due to the low detection rate of HCMV in semen - no more than 2.9% by the culture method [5,6] and 1.4-8.7% by PCR [5,7,8]. At the same time Neofytou et al. have detected HCMV DNA by PCR in the semen of 56.9% asymptomatic fertile and infertile patients [9].

There is a controversy over the effects of HCMV on the major parameters of sperm quality - concentration, motility and morphology of gametes. A correlation between high concentration of HCMV in ejaculate and a transient decrease in the spermatozoa motility have been established [10], and the concentration of sperm cells has been found to decrease in patients with HCMV in semen [11]. However, the majority of researchers have found no HCMV effect on sperm quality [3,7,12].

The issue of intragamete localization of HCMV is open to debate. The attempts to infect spermatozoa in vitro have been unsuccessful [13]. Investigation of interactions between HCMV and testicular cells is hampered by the possibility of autoimmune orchitis after biopsy. High species-specificity of HCMV impedes the investigation of processes occurring in human organism on animal models. In an attempt to overcome these difficulties we developed the model of HCMV infection in an organotypic culture of human testis. Using this model we demonstrated the possibility of intracellular HCMV localization in immature and mature male germ cells and HCMV influence on spermatogenesis.

## Materials and methods

### Patients

Semen samples were obtained from 138 men, including 91 infertile men (Group I) and 47 healthy donors enrolled in the sperm donor program (Group II). The informed consent was obtained from all patients.

### Clinical material

Semen samples were fractionated by gradient centrifugation with SupraSperm reagent (Origio, Jyllinge, Denmark) according to the World Health Organization Laboratory Manual for the Examination and Processing of Human Semen (2010). The fraction of motile spermatozoa (MS) was washed two times in 2 ml of Dulbecco modified Eagle Medium (DMEM; Paneko, Moscow, Russia) by centrifugation and used as described below.

### Virus and cell culture

HCMV AD 169 strain was provided by the Russian Federation State Collection of Viruses. The virus was propagated and titrated in human embryo lung fibroblasts (HEF).

### Rapid culture method (RCM)

RCM was used for detection of HCMV infectious activity in the samples. The material (0.2 ml) was injected into each well of 24-well culture plate (Costar, Washington DC, USA) with HEF confluent monolayer, incubated for 1 h at 37°C in an atmosphere of 95% air/5% CO_2_. The cells were washed 2 times in serum-free culture medium, incubated for 48 h in 1 ml of DMEM with 2% fetal calf serum (FCS; Gibco, Carlsbad, CA, USA), washed 2 times in PBS and fixed in cold methanol. HCMV was identified by immunoperoxidase staining with monoclonal antibody (Mab) against HCMV pp65 protein (DAKO, Glostrup, Denmark). Immunolabeled cells were calculated in an inverse light microscope LABOVEWRT FS (Leitz, Oberkochen, Germany).

### HCMV DNA detection and quantification by real-time PCR

HCMV DNA extraction was performed from 200 μl of samples using QIAamp DNA mini kit (QIAGEN, Hilden, Germany) according to the manufacturer's protocol. In brief, 200 μl of sample and 10 μl of the STI-87 positive internal control (PIC; Interlabservice, Moscow, Russia) were added to 200 μl of AL-buffer and heated at 56°C for 15 min. 200 μl of 96% ethanol was added, applied to columns and washed as per the manufacturer's instruction with the final elution in 200 μl of the kit AE-buffer preheated to 50°C. Real-time PCR was performed using the Amplisense CMV-screen/monitor-FL kit (Interlabservice) according to the manufacturer's protocol. Real-time amplification was carried out using 10 μl DNA eluate combined with 10 μl PCR-mix-1-FL and 5 μl PCR-mix-2-FL using Rotor-Gene 6000 Instrument (Corbett Research, Doncaster, Australia) with the following cycling parameters: pre-denaturation at 95°C for 15 min, 95°C for 5 s, 60°C for 20 s and 72°C for 15 s for 45 cycles. Data acquisition was performed in both JOE/Yellow (for HCMV DNA) and ROX/Orange (for the STI-87 PIC) channels during the annealing (60°C) stage. For quantification of HCMV DNA two standard positive sample KSG1 (10^4 ^copies per reaction mixture) and KGS2 (10^2 ^copies per reaction mixture) (Interlabservice) were included in the run. Calculations of *Ct*, preparation of standard curve and quantification of DNA in each sample were performed by Rotor-Gene Operating Software, version 1.8 (Corbett Research).

### PCR in situ

Washed-off MS were transferred to glass slides, centrifuged for 5 min at 1500 rpm in Cytospin 4 (Thermo Electron, Waltham, USA), air dried, fixed in 10% formaldehyde for 4 h and washed twice in 0.05 M Tris-HCl. The preparations were then incubated with proteinase K (DAKO) for 30 min at 37°C. Amplification with biotinilated primers (Gentech, Moscow, Russia) was carried out using T1 cycler (Biometra, Goettingen, Germany). Viral DNA was detected with the biotin-streptavidin-peroxidase complex (DAKO) and diaminobenzidene (Sigma-Aldrich, St Louis, MO, USA). The proportion of sperm cells containing HCMV DNA was calculated after analysis of at least 2000 cells.

### Spermiological and quantitative karyological analysis

Spermiological analysis was performed according to the World Health Organization Laboratory Manual for the Examination and Processing of Human Semen (2010). The immature germ cells (IGC) in sperm samples were identified by morphological criterions under a light microscope BX51 (Olympus, Tokyo, Japan). At least 200-300 IGC were calculated in each slide. The proportions of spermatids and primary spermatocytes at early stages (leptotene, zygotene, pachytene and diplotene) and cells that could not be identified (classified as unidentified and/or degenerated) were calculated as described previously [14].

### Organotypic culture of human testis explants

The procedures followed were in accordance with ethical standards of the Helsinki Declaration and were approved by the local Ethics Committee of the D.I. Ivanovsky Institute of Virology of Ministry of Health and Social Development of Russian Federation; informed consent was obtained from all patients. Testis samples from 3 patients with prostate cancer (62, 65 and 67 years old) were transported in fresh medium on ice immediately following orchidectomy. Testicular tissues were carefully dissected with scissors into 3 mm^3 ^fragments. In each well of a six-well plate, two fragments were placed onto a permeable membrane insert (Falcon Labware, Mt Pritchard, NSW, Australia) and incubated at the interface between air and 2 ml of DMEM with 10% FCS (Gibco), 1 mmol/l sodium piruvate, 100 ng/ml vitamin A, 50 ng/ml vitamin C and 200 ng/ml vitamin E (all from Sigma-Aldrich), 4 mmol/l glutamine, 10 μg/ml insulin, 5 μg/ml transferrin and 50 μg/ml gentamycin (all from Paneko) at 37°C in an atmosphere of 95% air/5% CO_2_. Culture medium was replaced every other day.

### HCMV-infection of testicular explants

Fragments were incubated with 0.025 ml of HCMV inoculate for 1 h at 37°C. Multiplicity of infection (MOI) was 0.0001-0.001 plaque forming units (PFU) per cell. Control uninfected cultures were incubated in DMEM under the same conditions. Then explants were washed three times in 1 ml DMEM and the culture was established for up to 14 days as described above. Viral load was estimated every other day in culture medium by PCR and RCM starting from Day 2. Three explants were analyzed in each point.

### Light microscopy

For histological analysis, testicular explants (3 mm^3^) were fixed in neutral buffered 10% formaldehyde for 24 h at 4°C, dehydrated in a series of graduated ethanol concentrations (70%, 96% and 100%), embedded in paraffin, sectioned at 4.0 μm and stained with Caracci's hematoxylin (BisVitrum, S.-Peterburg, Russia) for examination. The following morphological criteria were used for analysis of germ cells viability and explants architecture: cell number, cell size and location, signs of apoptosis, and histological characteristics of seminiferous tubules, including the basal membrane structure.

### Immunostaining

To reveal HCMV proteins in the testis explants immunostaining with Mab to HCMV pp65 was performed on formaldehyde-fixed, paraffin-embedded tissues. Antigen retrieval was performed as follows: deparaffinized and dehydrated sections were treated for 20 min at 750 Wt in microwave oven (Sanyo, Moriguchi, Osaka, Japan) in 10 mM citrate buffer (pH = 6.0, DAKO) and then washed in 0.05 mol/l phosphate-buffer saline (PBS, pH 7.6; Gibco). Endogenous peroxidase was inactivated in deparaffinized sections by 5-min treatment in PBS with 3% H_2_O_2_. Slides were processed by PBS supplemented with 2% bovine serum albumin (BSA, Sigma-Aldrich) to block the non-specific sites, before overnight incubation at 4°C with the Mab to HCMV pp65 (0.02 ug/ml) (DAKO) diluted in PBS with 1% BSA. Next steps were performed at room temperature. Reagents from UltraVision LP Large Volume Detection System kit (Thermo Scientific, Fremont, USA) were added according to the manufacturer's protocol after washing 4 times in PBS. The following substrate was used: 0.5 mg/ml diaminobenzidine (Sigma-Aldrich) in 0.05M TRIS-HCL, pH 8.0 with 3% H_2_O_2_. Sections were prepared immediately after tissue dissection and on days 2, 4, 7 and 14 after introducing in culture. Stained cells were identified and photographed with a BX51 microscope coupled to a digital macro camera U-CMAD3 (Olympus).

### Transmission electron microscopy (TEM)

TEM was performed immediately after tissue dissection and on days 2, 4, 7 and 14 of culturing. Two infected and two uninfected explants were analyzed in each point. The explants were fixed in 2.5% glutaraldehyde in 0.1 M buffered sodium cacodylate (pH 7.4) for 24 h at 4°C, postfixed in 1% OsO_4 _in 0.1 M sodium cacodylate buffer at room temperature for 40 min, then dehydrated in gradual ethanol concentrations (70%, 96% and 100%) and, subsequently, submitted to progressive impregnations in epon resin (Sigma-Aldrich). Polymerization was carried out at 60°C for 48 h. Ultrathin sections were cut in an ultratome III (LKB, Bromma, Sweden), stained with uranyl acetate and lead citrate (Sigma-Aldrich) and examined in a JEM-100 S electron microscope (JEOL, Tokyo, Japan) at 80 kV.

### Statistics and data analysis

The analysis was performed in StatXact 8 (Cytel, Cambridge, MA, USA) using Student's paired *t*-test, χ^2 ^test and Mann-Whitney test. P < 0.05 was considered statistically significant.

## Results

### HCMV detection in motile spermatozoa

We have identified HCMV in the fraction of motile spermatozoa by several methods (Table 1). The frequency of HCMV DNA detection by PCR was higher than frequency of infectious activity by RCM (p = 0.006) but without significant differences between groups irrespective of method. The percentage of HCMV-infected cells determined by PCR in situ was 1.7% in average (maximum 15%) among infertile men and 0.5% in average (maximum 5%) among healthy men (Table 1). In spite of a 3-fold increase in the number of infected cells in Group I comparing with Group II, this difference was not significant (p > 0.05).

**Table 1:** Detection of HCMV in the fraction of motile spermatozoa

Patients	Frequency of HCMV detection by		Percentage of spermatozoa containing HCMV DNA by PCR in situ, median % [min, max]
	rapid culture method	PCR	
Group I (Infertile)	3/91^a^(3.3%)	10/91 ^b ^(11%)	1.7 ^c ^[0.25; 15]

Group II (Fertile)	1/47 ^a ^(2.1%)	7/47 ^b ^(15%)	0.5 ^c ^[0.35; 5]

Groups I and II	4/138 (2.9%)	17/138 (12.3%)	1.5 [0.25; 15]

^a, b ^p > 0.05 (χ^2 ^test)

^c ^p > 0.05 (Mann-Whitney test)

### Spermiological and quantitative karyological analysis of HCMV-infected sperm

According to the results of HCMV detection in sperm, all samples were divided into two groups: with and without HCMV infection. Each group included infertile patients and sperm donors. A comparative spermiological analysis in these groups did not reveal any viral effect on the concentration of sperm cells (50.6 × 10^6 ^cells/ml vs. 69 × 10^6 ^cells/ml, p > 0.05), the percentage of motile spermatozoa (20% vs. 12.7%, p > 0.05) and morphologically normal germ cells (9% vs. 20%, p > 0.05). Quantitative karyological investigation enabled us to assess viral effect on spermatogenesis without invasive intervention. The number of unidentified and/or degenerated germ cells in HCMV-infected semen samples was higher (p < 0.05) while the population of spermatids was decreased (p < 0.05) comparing with uninfected samples (Table 2).

**Table 2:** Analysis of immature germ cells population in the HCMV-infected sperm

Patients	Spermatocytes, median %			Spermatides, median %	Unidentified/degenerative cells, median %
			
	Leptotene, zygotene	Pachytene	Diplotene		
Uninfected (n = 25)	3.3	0	0.43	86.5^a^	7.6 ^b^

HCMV-infected (n = 20)	2.9	0	0	78.3^a^	16.4 ^b^

^a, b^- p < 0.05 (Mann-Whitney test)

### HCMV-infection of testis organotypic culture

In order to study the effect of HCMV on spermatogenesis in more detail, we developed a model of HCMV infection in the human testis organotypic culture. It was demonstrated in preliminary experiments that general architecture of explants as well as viability of all germ cells preserved at least up to 14 days in culture.

Table 3 illustrates changes in HCMV markers which reflect the dynamics of viral infection. Due to high HCMV content in the inoculate (4.2 × 10^8 ^DNA copies/ml) the virus was not completely removed from the explants during washing procedure: after 12 h viral DNA content in the culture medium was 1.7 × 10^4 ^copies/ml. It gradually decreased within a 6-day period and increased starting from Day 8, indicating HCMV replication in the culture. Infectious activity reached the maximum on Day 12, while DNA HCMV being accumulated up to Day 14.

**Table 3:** HCMV markers in human testis organotypic culture

Method (viral load)	Days post infection						
	
	2	4	6	8	10	12	14
RCM (PFU/ml)	nd^a^	0	nd	2,5	nd	60	22,5

PCR (DNA copies/ml)	4730	3480	0	490	8600	8600	51900

*^a ^*nd - not determined

### HCMV detection in testicular cells

**Figure 1:**
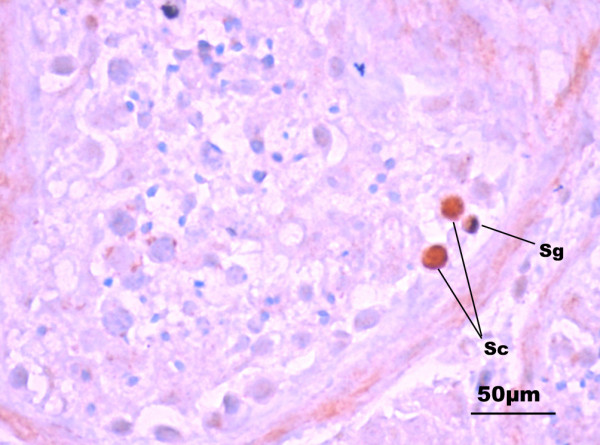
**HCMV detection in male germ cells on Day 14 post infection in testis organotypic culture**. Immunoreactivity with monoclonal antibodies specific to HCMV pp65 protein is shown on section of infected testis explants in organotypic culture on Day 14 post infection. Spermatogonia (sg) and spermatocytes (sc) are found to contain viral antigen.

**Figure 2:**
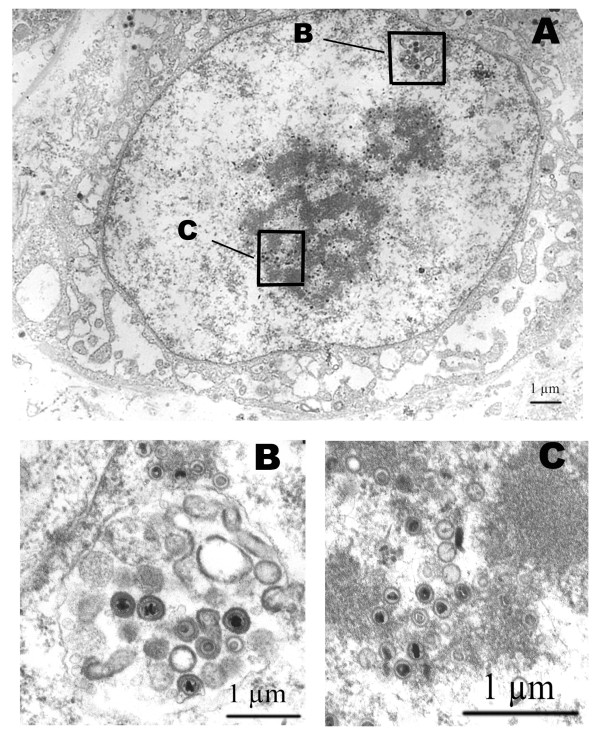
**HCMV in human spermatogonium on Day 14 post infection in testis organotypic culture**. Ultrathin section was obtained from HCMV-infected testis explants in organotypic culture on Day 14 post infection (A). Full and empty viral capsids (insert B) and virions (insert C) were demonstrated in the nucleus of spermatogonium.

On Day 4, viral antigens were identified by immunostaining in the interstitial cells (fibroblasts and Leydig cells) and in individual spermatids. Foci of infection were located in the superficial layers of explants which contacted with viral inoculate. By Day 7 HCMV spread into deeper layers and infected spermatocytes and spermatogonia were revealed. On Day 14, we observed fibroblasts with typical features of HCMV-infection: enlarged nuclei and huge inclusion bodies in cytoplasm. At later stages of infection, typical HCMV pp65 staining was detected in spermatids, spermatocytes, spermatogonia (Day 7) and individual spermatozoa (Day 14). Figure 1 illustrates the presence of HCMV protein in spermatogonium adjacent to the basal membrane of the tubule and in two large round cells identified as spermatocytes. Immunohistochemical data were confirmed by electron microscopy. A great number of virions contained empty and full capsides and electron-dense bodies were identified in germ cells. Figure 2 shows the spermatogonium with capsids in the nucleus and virions with typical herpes-virus morphology in transport vacuole.

### Decrease of the germ cells population in HCMV- infected testis explants

The effect of HCMV on spermatogenesis was studied histologically by comparing of infected and uninfected testis explants at different time in culture. The results for germ cell populations on Days 7 and 14 are summarized in Table 4. It was shown that the number of spermatogonia, spermatocytes, round and elongated spermatids decreased considerably starting from Day 7 of infection. It should be noted that there were no changes in the number of spermatozoa. By Day 14, changes in immature cell population were more pronounced, reflecting gradual destruction in testicular architecture, loosening and vacuolization of germinative epithelium.

**Table 4:** Quantitative analysis of immature and mature germ cells in HCMV-infected human testis organotypic culture

Germ cells	Organotypic culture in vitro, days					
	
	7			14		
	
	Uninfected	HCMV-infected	P-value	Uninfected	HCMV-infected	P-value
Spermatogonia	100^a^	53,8	<0.001^b^	82,2	36,8	<0.001

Spermatocytes	100	38,9	<0.001	51,5	18,7	<0.001

Round spermatids	104	37,7	<0.001	70,4	27,6	<0.001

Elongated spermatids	96	57,7	<0.001	65,7	42,5	<0.05

Spermatozoa	106,3	100	>0.05	106,3	100	>0.05

^a ^- mean quantity of cells, %; quantity of cells before introduction in culture was taken for 100%;

^b ^- Student's paired t-test.

## Discussion

The possibility of vertical transmission of herpes viruses with male gametes has been declared by several investigators [15,16]. In the first part of this work we studied intracellular HCMV localization in male gametes as a potential transmission vector of infection. HCMV DNA was found in 12.3% of sperm cells (mean for Groups I and II), and infectious virus - in 2.9% of all cases. The percentage of infected cells reached 15% in infertile patients and 5% in healthy donors, while the mean value in both groups was found to be 1.5%.

There is no direct data concerning the correlation of HCMV infection of human spermatozoa and miscarriages and fetal maldevelopment. The results obtained in animal experiments are controversial. According to Neighbour et al. mouse CMV produced no effect on fertilization and embryogenesis in mice [16]. At the same time inhibition of blastocyst formation after infection of two-cell embryos with mouse CMV was observed [17]. There is evidence that herpes virus infection of males plays a role in fetal loss in goats [18]. Statistical analysis has been used to evaluate the role of herpes viruses in human reproduction. The frequency of herpes simplex virus detection in sperm samples of partners of women with repeated miscarriages was higher than in the control (p < 0.05) [19]. A correlation between the presence of herpes viruses in ejaculate and negative outcome of pregnancy can be regarded as an indirect evidence for a vertical herpes virus transmission and associated pregnancy loss. The percentage of gametes carrying HCMV may serve as a criterion for estimation of the risk of vertical transmission of infection.

The ability of the virus to replicate in male germ cells was confirmed in the second part of the study using testis organotypic culture. Intensive viral accumulation occurred in the testicular interstitium, especially in fibroblasts where HCMV was detected starting from the Day 4 of infection. On Day 8, viral antigens were identified in spermatogonia and spermatocytes, and on Day 14 - in spermatozoa. Infection of germ cells was confirmed by electron microscopy.

Data obtained supposes that the presence of HCMV markers in the mature spermatozoa, which was demonstrated both in organotypic culture and in sperm samples, is a consequence of the precursor immature germ cells infection. In vivo it takes 14 days for the round spermatid differentiation into the spermatozoon [20], while in vitro this period was found to be much shorter [21,22]. These findings suggest that the presence of HCMV in spermatozoa at late stages of cultivation was due to differentiation of infected spermatids which had been already detected on the Day 4 of infection.

The association between HCMV infection and male infertility is discussed. In this work no difference between infertile and healthy men was found neither in the frequency of HCMV identification in sperm nor in the number of infected gametes. Routine semen analysis revealed no significant differences between infected and virus-free samples in concordance with previous reports [7,12]. Nevertheless quantitative karyological analysis demonstrated a decrease in the number of spermatids in sperm with simultaneous increase in number of unidentified/degenerated germ cells. These data correspond to the results of Moustafa et al. that the number of apoptotic cells is greater in sperm of infertile men in comparison with that of healthy donors [23]. Our results are also consistent with those of Wu et al. demonstrating an increase in number of apoptotic immature germ cells with chromatin pycnosis and vacuolation, damaged nuclear membrane, and apoptotic bodies in HCMV-infected semen samples [11].

Cultivation of testis explants in vitro allows one to examine the effects of various factors on spermatogenesis for at least two weeks. At the beginning of the second week post infection the following signs of viral replication were observed: the increase in viral DNA load and HCMV infectious activity with the spread of infection foci in the explants. The number of IGC decreased during the second week in vitro. This finding indicates that HCMV produces a specific lytic effect on germ cells at different stages of development, i.e., on spermatogonia, spermatocytes and spermatids.

The safety of spermatozoa population in infected culture raises the question of why HCMV has a deleterious effect on developing germ cells but not on mature cells. One of the possible explanations for such a difference is abortive HCMV infection of spermatozoa. This suggestion is supported by the fact that only empty capsids without electron-dense core (type A and type B) have been detected in mature sperm cells [24]. Our group and other researchers failed to identify filled capsids (type C) and enveloped virions into spermatozoa. By contrast, infected IGC contain all types of viral particles (Figure 2). A considerable loss of germ cells in an infected testis organotypic culture (Table 4) points to a lytic type of HCMV-infection in IGC. Current knowledge does not allow us to characterize exactly molecular mechanisms responsible for blocking HCMV morphogenesis in spermatozoa. One can suggest that the abortive nature of the infection is determined by the events occurring in a maturing gamete: 1) hypercondensation of chromatin with switching off majority of genes, including those that are necessary for viral replication; 2) loss of the cytoplasm with considerable part of replication machinery; 3) impaired nucleus-cytoplasm transport due to nuclear pore complex modification [25]. In sperm samples HCMV-infection of IGC resulted in decrease of spermatids and in increase of degenerative germ cells. At the same time neither concentration, nor sperm motility and morphology were found to be affected in infected sperm samples. We may suggest that in vivo the number of uninfected IGC is enough for producing a quantity of spermatozoa sufficient for fertilization. Moreover it should be noted that the major parameters of semen are highly variable in a population [26] and on the other hand, the frequency of HCMV detection and viral load in sperm is rather low. It means that a large group of patients would be required to reveal the HCMV effects on male fertility.

## Conclusions

HCMV was found in male germ cells both in sperm samples and in testis organotypic culture infected in vitro. Data obtained suppose that HCMV infects immature germ cells that develop to mature HCMV-carrying spermatozoa. The significant decrease in immature germ cells upon viral infection indicates that HCMV produces a direct gametotoxic effect and can contribute in male infertility.

## Abbreviations

BSA: bovine serum albumin; DMEM: Dulbecco modified Eagle medium; FCS: fetal calf serum; HCMV: human cytomegalovirus; HEF: human embryo lung fibroblasts; IGC: immature germ cells; Mab: monoclonal antibody; MOI: multiplicity of infection; MS: motile spermatozoa; PBS: phosphate-buffered saline; PFU: plaque forming units; PIC: positive internal control; RCM: rapid cultural method; TEM: transmission electron microscopy.

## Competing interests

The authors declare that they have no competing interests.

## Authors' contributions

VAN performed the TEM, analyzed data and drafted the paper. YAT performed cultural work. SAY and ASS performed clinical investigation of sperm donors and infertile patients. LFK and LVS performed quantitative karyological investigation and morphological analysis of testis organotypic culture. LEZ performed immunostaining. RRK and SVA performed PCR in situ and PCR-rt. AAK designed the study, analyzed data and edited manuscript. All authors read and approved the final manuscript.
